# A Consensus Statement on acromegaly therapeutic outcomes

**DOI:** 10.1038/s41574-018-0058-5

**Published:** 2018-07-26

**Authors:** Shlomo Melmed, Marcello D. Bronstein, Philippe Chanson, Anne Klibanski, Felipe F. Casanueva, John A. H. Wass, Christian J. Strasburger, Anton Luger, David R. Clemmons, Andrea Giustina

**Affiliations:** 1 Department of Medicine, Cedars-Sinai Medical Center, Los Angeles, CA USA; 2 Division of Endocrinology and Metabolism, Hospital das Clinicas, University of São Paulo, São Paulo, Brazil; 3 Assistance Publique-Hôpitaux de Paris, Service d’Endocrinologie et des Maladies de la Reproduction, Centre de Référence des Maladies Rares de l’Hypophyse, Hôpital Bicêtre, Paris, France; 4 UMR S-1185, Faculté de Médecine Paris-Sud, Université Paris-Sud, Université Paris-Saclay, Paris, France; 5 Department of Medicine, Massachusetts General Hospital, Boston, MA USA; 6 Department of Medicine, Santiago de Compostela University, Santiago de Compostela, Spain; 7 Department of Endocrinology, Churchill Hospital, Oxford, UK; 8 Department of Medicine, Charité Universitätsmedizin Campus Mitte, Berlin, Germany; 9 Division of Endocrinology and Metabolism, Medical University of Vienna, Vienna, Austria; 10 Department of Medicine, University of North Carolina, Chapel Hill, NC USA; 11 Department of Endocrinology and Metabolism, San Raffaele University Hospital Milan, Milan, Italy

**Keywords:** Growth disorders, Drug therapy, Pituitary tumours

## Abstract

The 11th Acromegaly Consensus Conference in April 2017 was convened to update recommendations on therapeutic outcomes for patients with acromegaly. Consensus guidelines on the medical management of acromegaly were last published in 2014; since then, new pharmacological agents have been developed and new approaches to treatment sequencing have been considered. Thirty-seven experts in the management of patients with acromegaly reviewed the current literature and assessed changes in drug approvals, clinical practice standards and clinical opinion. They considered current treatment outcome goals with a focus on the impact of current and emerging somatostatin receptor ligands, growth hormone receptor antagonists and dopamine agonists on biochemical, clinical, tumour mass and surgical outcomes. The participants discussed factors that would determine pharmacological choices as well as the proposed place of each agent in the guidelines. We present consensus recommendations highlighting how acromegaly management could be optimized in clinical practice.

## Abstract (web summary)

Acromegaly is usually caused by a pituitary adenoma that secretes growth hormone; treatment of acromegaly and its comorbidities is often complex. In this Consensus Statement, Shlomo Melmed and colleagues provide updated recommendations for the treatment of patients with acromegaly.

## Introduction

Acromegaly is caused by excess circulating levels of growth hormone (GH) and insulin-like growth factor 1 (IGF1), which typically result from a GH-secreting pituitary adenoma^1^. Patients exhibit characteristic acral and soft tissue overgrowth (particularly in the face and hands), arthritis, jaw overbite, respiratory obstruction, hypertension and headache, as well as visual disturbances and cranial nerve palsy from tumour mass effects^2^. Metabolic dysfunction, including insulin resistance and elevated HbA_1c_, increases the risk of diabetes mellitus and cardiovascular-related morbidity and mortality^3^. Treatment of patients with acromegaly is aimed at normalizing GH and/or IGF1 levels to ameliorate signs and symptoms of the disease^2,4,5^ and reduce excess mortality^6–8^.

Long-term biochemical control is achieved in fewer than 65% of patients following surgical resection of the tumour despite the use of novel surgical approaches^9–15^, and only approximately half of patients treated with medical therapy achieve control of IGF1 levels^16–19^. Radiation therapy remains an option in patients with persistently active disease, but rates of control and safety have only marginally improved with the use of stereotactic radiosurgery instead of conventional fractionated radiotherapy^20^. Management of acromegaly and the comorbidities of the disorder is complex and requires a comprehensive approach coordinated by a multidisciplinary team of physicians who are experts in the treatment of pituitary tumours^21^.

In April 2017, the Acromegaly Consensus Group convened to update the most recent consensus guidelines on the medical management of acromegaly, which were published in 2014 (ref.^4^). Since that publication, new pharmacological agents have been developed and new approaches to treatment sequencing have been considered. Thirty-seven experts in acromegaly management (Box 1) reviewed the current literature and assessed changes in drug approvals, clinical practice standards and clinical opinion since the 2014 consensus publication. Discussions focused on treatment outcome goals; effects of pharmacological agents on biochemical, clinical, tumour volume and surgical outcomes; factors determining pharmacological choices; and the proposed place of available pharmacological agents in the guidelines. Updated consensus recommendations on therapeutic outcomes for patients with acromegaly were graded using the Grading of Recommendations Assessment, Development and Evaluation system^22,23^ (Box 2), and the key recommendations are presented in Box 3. Key changes from the 2014 consensus recommendations are presented in Table 1.

**Table 1:** Key changes from the 2014 to the 2018 consensus recommendations

Strategy	2014 consensus recommendation^4^	2018 consensus recommendation
Management approach	Not addressed	Multidisciplinary team approach at a pituitary tumour centre of excellence, where possible
Defining and monitoring biochemical control	GH nadir <1 µg/l after OGTT on sensitive assays	• GH nadir < 0.4 µg/l after OGTT using ultrasensitive assays • Wait at least 12 weeks after surgery to assess IGF1 levels (delayed decline versus persistent postoperative GH) • Do not measure GH in patients receiving pegvisomant (levels remain elevated)
First-line medical therapy in patients with persistent disease after surgery	• SRL (octreotide LAR or lanreotide autogel) • Cabergoline if IGF1 <2 times the upper limit of normal	• First-generation SRL (octreotide LAR or lanreotide autogel) • Cabergoline if IGF1 <2.5 times the upper limit of normal
Second-line medical therapy if first-generation SRL is not successful in normalizing IGF1	Partial response: • Increase SRL dose or decrease dose interval • Add pegvisomant to SRL • Add cabergoline to SRL Minimal or no response: • Switch to pegvisomant	Partial response: • Increase first-generation SRL dose and/or increase dose frequency of lanreotide autogel • Add cabergoline to SRL if IGF1 is moderately elevated Minimal or no response and tumour concern: • Switch to pasireotide LAR Minimal or no response and impaired glucose metabolism: • Switch to pegvisomant Minimal or no response, tumour concern and impaired glucose metabolism: • Add pegvisomant to first-generation SRL
Therapy if biochemical control is not achieved after second-line therapy	• Optimize pegvisomant dose • Switch to pegvisomant plus dopamine agonist • Add dopamine agonist to SRL	• Stereotactic radiosurgery or surgical intervention (or reintervention) • Temozolomide for unusually aggressive or proven malignant tumours (in close cooperation with a neuro-oncologist)
Use of clinical outcome instruments	Not addressed	• Objective tools (SAGIT and ACRODAT) can be used to assess and monitor indicators of disease activity • Patient quality of life questionnaires (AcroQoL) are probably of limited value

ACRODAT, Acromegaly Disease Activity Tool; GH, growth hormone; IGF1, insulin-like growth factor 1; LAR, long-acting release; OGTT, oral glucose tolerance test; SAGIT, Signs and symptoms, Associated comorbidities, GH levels, IGF1 levels and Tumour profile; SRL, somatostatin receptor ligand.

Box 1 11th Acromegaly Consensus Conference participants Ariel Barkan (USA), Albert Beckers (Belgium), Nienke Biermasz (Netherlands), Beverly Biller (USA), Cesar Boguszewski (Brazil), Marek Bolanowski (Poland), Marcello Bronstein (Brazil), Felipe Casanueva (Spain), Philippe Chanson (France), David Clemmons (USA), Annamaria Colao (Italy), Diego Ferone (Italy), Maria Fleseriu (USA), Monica Gadelha (Brazil), Ezio Ghigo (Italy), Andrea Giustina (Italy), Mark Gurnell (UK), Anthony Heaney (USA), Andrew Hoffman (USA), Laurence Katznelson (USA), Fahrettin Kelestimur (Turkey), Anne Klibanski (USA), Steven Lamberts (Netherlands), Anton Luger (Austria), Gherardo Mazziotti (Italy), Shlomo Melmed (USA), Pietro Mortini (Italy), Marco Losa (Italy), Sebastian Neggers (Netherlands), Stephan Petersenn (Germany), Roberto Salvatori (USA), Christian Strasburger (Germany), Peter Trainer (UK), Stylianos Tsagarakis (Greece), John Wass (UK), Susan Webb (Spain) and Maria Chiara Zatelli (Italy).

Box 2 Grading of evidence and recommendations **Grading the evidence**

- Very low quality (VLQ): expert opinion supported by one or few small uncontrolled studies
- Low quality (LQ): supported by large series of small uncontrolled studies
- Moderate quality (MQ): supported by one or few large uncontrolled studies or meta-analyses
- High quality (HQ): supported by controlled studies or large series of large uncontrolled studies with sufficiently long follow-up

**Grading the recommendations**

- Discretionary recommendation (DR): based on VLQ or LQ evidence
- Strong recommendation (SR): based on MQ or HQ evidence

Adapted from ref.^4^, Macmillan Publishers Ltd.

Box 3 Key 2018 consensus recommendations

- We recommend patients be treated at pituitary tumour centres of excellence, where possible, to receive the best and most cost-effective care.
- Surgical resection of the pituitary adenoma by an experienced neurosurgeon is recommended where possible and represents the best opportunity for cure.
- Medical therapy is recommended for patients with persistent disease despite surgical resection of the adenoma as well as patients in whom surgery is not appropriate.
- For patients with persistent disease after surgery, a first-generation long-acting somatostatin receptor ligand (SRL) is recommended as first-line therapy.
- If clinically relevant residual tumour that is unsuitable for resection is present, patients not adequately controlled on first-generation SRLs could be considered for switching to pasireotide long-acting release.
- If there is pre-existing clinically relevant impaired glucose metabolism, patients not adequately controlled on first-generation SRLs should be switched to pegvisomant.

## Methods

Meeting participants were assigned specific topics related to acromegaly treatment and outcomes. Literature searches were conducted using PubMed for English-language papers published between April 2013 and March 2017. Search terms included “acromegaly” and terms associated with each topic: “biochemical outcomes”, “tumour volume”, “clinical symptoms”, “somatostatin receptor ligand”, “dopamine agonist”, “GH receptor antagonist”, “estrogen”, “selective estrogen receptor modulator”, “mortality”, “complications”, “surgical outcomes” and “guidelines”. After a brief presentation on each topic to the entire group, participants were divided into subgroups for further discussion of the topic and reported their findings to the entire group. Participants developed consensus recommendations on the basis of all presentations, discussions and reports. All participants then voted on each recommendation. After the meeting, the Scientific Committee graded the evidence supporting the recommendations, and then graded the consensus recommendations on the basis of the quality of evidence (Box 2).

## Treatment outcome goals

### Biochemical outcomes

Excess GH and/or IGF1 in patients with acromegaly leads to metabolic, cardiovascular and musculoskeletal comorbidities, which, in turn, increase mortality as a result of cardiovascular, cerebrovascular and respiratory abnormalities^1,7^. Treatment is aimed at normalizing IGF1 levels, as doing so usually reflects adequate disease control, decreases risk of developing complications from comorbidities^24^ and might also reduce excess mortality^6,25^. However, large variability exists between the different IGF1 assays (moderate quality (MQ)). Pre-analytical and analytical factors can confound results^26^, and differences in normative data and reference ranges make it difficult to compare results across assays^27,28^. It is therefore recommended that, whenever possible, endocrinologists use the same assay when monitoring IGF1 levels over time and that the selected assays adhere to accepted performance standards^26^ (strong recommendation (SR)). Newer techniques, such as mass spectrometry^29^, might offer an improvement over older immunoassays but might not be routinely available.

GH nadir levels <1 µg/l after an oral glucose tolerance test (OGTT) were first defined by our Consensus Group as reflective of postoperative cure in 2000 (ref.^30^). Data from large observational studies continue to show improved long-term outcomes and reduced mortality in patients who achieve GH <1 µg/l after surgery^11,25,31^ (MQ). When ultrasensitive GH assays are available, we recommend an OGTT GH cut-off of 0.4 µg/l (SR). Although this lower cut-off might not further improve metabolic outcomes^32^, nor markedly influence the percentage of patients who achieve biochemical remission^31^, it is better suited to the lower limits of detection of the newer assays^33–35^. GH nadir levels during an OGTT are also affected by factors such as patient age, BMI, sex and oestrogen use, and we recommend that these factors are considered when interpreting results of this test^26,36^ (discretionary recommendation (DR)).

The hypothalamic-controlled episodic pattern of GH secretion that is seen in healthy individuals is retained in patients with acromegaly^37^, but might not correlate with levels of IGF1 in patients who have been treated with medical therapy^38^ (low quality (LQ)). We recommend monitoring biochemical control by measuring both GH and IGF1 levels (SR). However, we recommend that normalizing levels of IGF1 is a key goal, as it is the best reflection of disease control^38^ (DR). As GH levels remain elevated with pegvisomant therapy, measuring GH in patients receiving pegvisomant should not be done^18^ (high quality (HQ)). Monitoring of GH levels can be used to directly monitor tumour activity^39^ (very low quality (VLQ)), but we recommend waiting at least 12 weeks after surgery to assess IGF1 levels, as the postoperative decline in IGF1 levels can be delayed compared with that of GH levels^11,40^ (SR). Discordant reported IGF1 and GH values have been observed in patients following surgery as well as in those treated with somatostatin receptor ligands (SRLs)^41,42^ (MQ), which is probably the result of discrepancies in the assays used (MQ) and/or of biological factors, such as sex, glucose metabolism and GH receptor polymorphism, affecting results^43,44^ (VLQ). As the clinical importance of such a finding remains to be established, performing an OGTT in patients treated with an SRL is not likely to be clinically useful^38^.

### Tumour volume

Reducing tumour size and preventing further tumour growth are clinically relevant goals for patients with acromegaly and macroadenomas (≥10 mm), as the presence of these larger tumours is independently associated with poor clinical outcomes^45^. Most current series evaluating tumour response to SRL therapy use a volume reduction cut-off of 20–25% to define significant reduction (LQ), as it seems unlikely that lower thresholds could be determined owing to methodological variability. However, accurately measuring volume in clinical practice might be hampered by technical differences in methods, tumour shape and intra-observer inconsistencies^46^ (VLQ). For routine measurements in standard clinical practice, we recommend that reduction in a single tumour dimension, such as diameter, rather than tumour volume, might be simpler to measure and is sufficient to assess meaningful mass change^46,47^ (DR). T2-weighted MRI hypointensity at diagnosis predicts tumour shrinkage in patients receiving SRL therapy (MQ), and we recommend that this factor might be a useful marker of tumour responsiveness^48^ (DR).

### Clinical symptoms

Prevention and management of disease-associated symptoms and comorbidities are critical to improving clinical outcomes in patients with acromegaly^49^. Cardiovascular and respiratory effects are major causes of morbidity and mortality^6,8,25^ (HQ), and impaired glucose metabolism further contributes to increased cardiovascular risk^50,51^ (HQ). We recommend assessing and aggressively managing disease-associated comorbidities, specifically hypertension and cardiac hypertrophy, diabetes mellitus and glucose intolerance, sleep apnoea and osteopathy (SR). In patients with uncontrolled disease, these comorbidities should be aggressively managed to prevent excess mortality. When GH and/or IGF1 levels are controlled, regular 6-month follow-up is prudent. Clinician-reported outcome instruments such as SAGIT (Signs and symptoms, Associated comorbidities, GH levels, IGF1 levels and Tumour profile) and ACRODAT (Acromegaly Disease Activity Tool) provide objective measurements of acromegaly signs and symptoms, comorbidities, tumour profile, GH levels and IGF1 levels (VLQ), and we recommend that they can be used to assess and monitor indicators of disease activity^52,53^ (DR). Patient-reported health-related quality of life should also be considered. However, results from the acromegaly-specific questionnaire AcroQoL do not consistently correlate with biochemical control^54–56^, and interpretation of discordant biochemical and quality of life results remains unclear. Routine use of this tool in clinical practice is probably of limited value (DR).

### Pituitary tumour centres of excellence

Treatment of acromegaly is best accomplished by a multidisciplinary team of experts meeting together in person or virtually^21^ (MQ). With this structure, termed a pituitary tumour centre of excellence, in addition to neurosurgeons expert in transsphenoidal pituitary surgery and endocrinologists well versed in the full spectrum of medical therapies, the management team should comprise neuroradiologists well trained in pituitary and parasellar imaging; neuropathologists with expertise in molecular analysis; and radiation oncologists with specific knowledge in treating intracranial tumours (LQ). The availability of skilled nurses experienced in relevant pituitary therapies and patient education is important. We recommend that patients are treated at pituitary tumour centres of excellence to receive the best and most cost-effective care (SR). However, as patient access to such centres might be limited^21^, consensus recommendations are provided to optimize acromegaly therapeutic outcomes in routine clinical practice.

## Biochemical results of medical therapy

Medical therapy is recommended for patients with persistent disease despite surgical resection of the adenoma as well as for patients in whom surgery is not appropriate (SR). The SRLs octreotide, lanreotide and pasireotide, as well as the dopamine agonist cabergoline, bind cognate receptors in the adenoma and suppress GH secretion; the GH antagonist pegvisomant blocks GH action in the periphery and blocks generation of IGF1 (refs^57–59^).

### Somatostatin receptor ligands

#### First-generation somatostatin receptor ligands

Biochemical control rates of approximately 55% have been reported with the first-generation SRLs octreotide and lanretotide^60^; however, data from rigorously conducted trials using currently available long-acting formulations show lower rates of 25–45%^16,17,19,61^ (MQ). As patient selection bias, initial IGF1 levels, previous surgery, adverse effects and treatment compliance can all impact the likelihood of achieving biochemical control, in practice, biochemical response to first-generation SRLs is likely to be higher than that observed in trials published in the past 10 years but lower than in earlier trials (LQ)^62^. Octreotide long-acting release (LAR) is administered once monthly by intramuscular injection; lanreotide autogel is administered once monthly subcutaneously by the patient, their caregiver or a health-care provider. As efficacy rates are similar for the two agents^19,60^, preference for route of delivery and/or associated cost might influence treatment choice^63^ (VLQ).

**Fig. 1:**
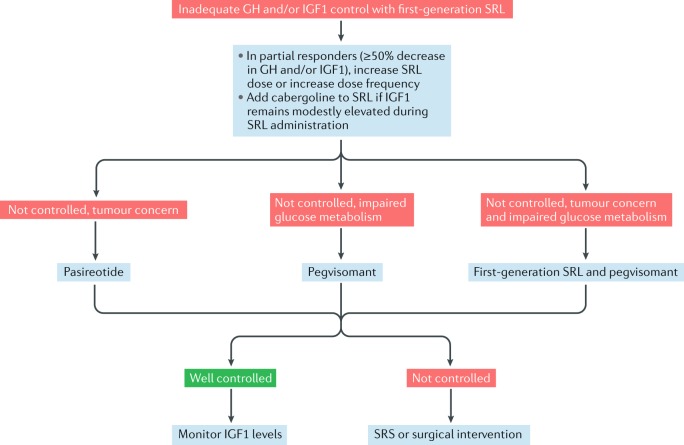
A proposed algorithm for the treatment of acromegaly in patients inadequately controlled with first-generation somatostatin receptor ligands lanreotide autogel and octreotide long-acting release. In partial responders (≥50% decrease in growth hormone (GH) and/or insulin-like growth factor 1 (IGF1)), increase somatostatin receptor ligand (SRL) dose and/or dose frequency. If IGF1 remains modestly elevated during SRL administration, add cabergoline to SRL. If disease control is not achieved, patients should be switched to the second-generation SRL pasireotide if there is clinically relevant residual tumour on imaging and/or clinical concern of tumour growth (tumour concern). Patients with impaired glucose tolerance should be switched to the GH antagonist pegvisomant. Patients with impaired glucose tolerance and tumour concern should be treated with a combination of a first-generation SRL and pegvisomant. Those who remain uncontrolled despite second-line medical therapy should be considered for stereotactic radiosurgery (SRS) or surgical intervention.

Studies have shown that higher doses of octreotide LAR (60 mg every 28 days) as well as higher doses (180 mg every 28 days) and more frequent dosing (120 mg every 21 days) of lanreotide autogel can improve biochemical control rates in patients who are inadequately controlled on standard doses but are responsive to SRL therapy^64,65^ (MQ). The maximal dosing of first-generation SRLs remains to be clarified. Careful patient selection, including considering degree of responsiveness to standard dosing, baseline IGF1 levels and treatment adverse effect profiles, is recommended before implementing such strategies (DR) (Fig. 1).

#### Second-generation somatostatin receptor ligands

Biochemical control rates with pasireotide LAR are higher than those achieved with octreotide LAR in patients who have not previously been treated with an SRL^61^ (MQ). However, normalized levels of IGF1 are still achieved in fewer than half of patients treated with pasireotide LAR, and nearly 70% of patients treated with pasireotide LAR exhibited hyperglycaemia-associated adverse effects^66^ (MQ). As patients with inadequately controlled disease on octreotide LAR or lanreotide autogel show improved biochemical control after switching to pasireotide LAR^66^, we recommend pasireotide LAR be considered a second-line therapy (SR) (Fig. 1). Elevated HbA_1c_ and fasting plasma levels of glucose at baseline are strong predictors for developing hyperglycaemia during treatment with pasireotide LAR^67^ (MQ). We recommend that patients considered for treatment with pasireotide LAR should be carefully screened and monitored for glycaemic adverse effects (SR), and pasireotide LAR should preferably be used in those with normal glucose tolerance. Blood levels of glucose should be monitored weekly for the first 3 months of treatment and in the first 4–6 weeks after dose increases. Monitoring should continue throughout treatment, as clinically appropriate.

#### Somatostatin receptor ligands in development

New formulations of SRLs are currently in clinical development, including oral octreotide capsules, parenteral octreotide bound in a liquid crystal mix and a parenteral multi-ligand SRL with high selectivity for GH suppression^68,69^. A phase III study of oral octreotide in patients well controlled on octreotide LAR showed that biochemical control rates were maintained after switching to oral octreotide, and patient acceptability and compliance were improved owing to route of administration^70^ (LQ). Additional studies with oral octreotide are currently underway^71,72^.

### Dopamine agonist

Cabergoline monotherapy results in biochemical control rates of approximately 35%; similar benefits have also been seen with the addition of cabergoline to an SRL in patients with inadequate control on SRL therapy^73^ (LQ). However, the benefits are largely limited to patients with mildly elevated levels of IGF1 at baseline, with the greatest benefit seen in those with IGF1 levels ≤1.5 times the upper limit of normal (MQ). We recommend that cabergoline should therefore be considered as a first-line medical therapy or as an addition to first-generation SRL in patients with IGF1 levels <2.5 times the upper limit of normal (DR).

### GH receptor antagonist

Pegvisomant monotherapy administered as second-line therapy yields biochemical control rates of 90% or more in clinical trials^18,74^ (HQ) and closer to 60% in real-world surveillance studies^75,76^ (MQ). This difference is probably primarily attributable to differences in doses, as patients in clinical practice are less likely to be uptitrated to the maximum dose despite higher efficacy rates being seen at higher doses^77^ (VLQ). Pegvisomant is approved for use at doses ranging from 10 mg per day to 30 mg per day, and we recommend that the daily dose should be increased to the recommended highest dose as needed (SR). Patient-specific factors such as age and BMI have been identified as predictive of the dose of pegvisomant that is required for normalization of IGF1 levels^78,79^ (LQ), but we recommend that physicians should regularly monitor IGF1 levels throughout therapy to determine whether normalization can be achieved by adapting the dose regimen^59^ (SR). Surveillance studies show that high doses of up to 60 mg per day have been used in patients with persistently elevated IGF1 levels^80^; however, use of doses above 30 mg per day is not approved, has not been prospectively studied and therefore is not recommended in clinical practice (DR).

Similarly, pegvisomant has shown high efficacy rates when given in combination with an SRL and delivered once or twice weekly^81,82^ (MQ) and might show continued effectiveness after discontinuing the SRL^83^ (LQ). Analysis of surveillance data suggests a biochemical control rate of approximately 75% in patients treated with pegvisomant monotherapy as first-line therapy^84^, but prospective data are lacking (VLQ).

### Oestrogens and SERMs

Oestrogens and selective oestrogen receptor modulators (SERMs) reduce levels of IGF1 in patients with acromegaly when used alone or in combination with an SRL or cabergoline^85^ (VLQ). SERMs might have an additional benefit in men with acromegaly and hypogonadism, as these agents also increase levels of testosterone^86,87^ (VLQ). However, as published evidence is limited, optimal use of these agents remains undetermined, and sex-specific adverse effects should also be considered.

## Clinical outcomes of medical therapy

Although biochemical control is the primary aim of acromegaly treatment, physicians should also consider the effect of therapy on disease-related morbidity and mortality. As a result, physicians should implement strategies to prevent, address and manage acromegaly complications.

### Mortality

The increased mortality that is associated with acromegaly is largely ameliorated in patients with adequately controlled disease, who have mortality similar to that of the general population^6,7^ (MQ). In addition, patients followed up in the long term show a shift away from cardiovascular disease to cancer as a leading cause of death^25,88,89^ (LQ). A continuum of benefit results from normalizing GH and IGF1 levels, leading to improved outcomes of disease-related comorbidities and reduced mortality risk^25^. However, the effects of specific treatment modalities on mortality in patients not cured with surgery are unclear. Data linking conventional radiotherapy with increased mortality might not apply to stereotactic radiosurgery given the potential improvements in treatment outcomes^20,90,91^ (VLQ), but effects on mortality have not been sufficiently investigated. Data on the long-term impact of medical therapy on all-cause mortality are few and inconclusive.

### Complications

Cardiomyopathy, hypertension, valvular disease, arrhythmias and sodium and fluid retention leading to expanded extracellular fluid volume are seen in more than 60% of patients with acromegaly and are a major cause of disease-associated morbidity and mortality^3,49^ (HQ). Surgery, SRLs and pegvisomant can all improve left ventricular hypertrophy in patients who achieve biochemical control^92–94^ (MQ); improvement in hypertension and arrhythmias has also been shown in patients effectively treated with medical therapy^93,95^ (LQ). A study published in 2012 that examined potential adverse valvular effects associated with high-dose cabergoline in other diseases found no such effect in patients with acromegaly, which is reassuring^96^. As cardiac comorbidities might persist despite biochemical control of acromegaly, regular monitoring of patients is recommended (SR).

Vertebral fractures have been observed in up to 60% of patients with acromegaly^97–99^ (MQ). These fractures can be present despite disease control^100,101^ and are frequently asymptomatic^97^. Normal BMD on dual X-ray absorptiometry might offer false reassurance, as BMD does not predict fracture risk in patients with acromegaly^97,98,101^ (MQ). Bone turnover is probably a better indicator of bone quality^101,102^ (LQ), and proactive evaluations of vertebral fractures with the morphometric approach are recommended at diagnosis and annually thereafter^103^ (SR). Assessment of bone microarchitecture in men with acromegaly has shown that alterations in both cortical and trabecular bone occur, which further corroborates the limitations of using areal BMD to assess fracture risk in these patients^104^.

Soft tissue and bony craniofacial overgrowth result in considerable airway obstruction and respiratory complications in at least 25% of patients with acromegaly and might not be reversible despite the achievement of adequate biochemical control^49^ (MQ). We recommend that screening questionnaires for obstructive sleep apnoea are used in clinical practice, with sleep studies ordered as needed to confirm the diagnosis (SR). We also recommend that management strategies such as continuous positive airway pressure therapy should be considered for patients with persistent symptoms independent of acromegaly treatment^105^ (DR).

Impaired glucose metabolism and diabetes mellitus, which are present in up to half of patients with acromegaly, are infrequently affected by treatment with first-generation SRLs^106^ (MQ) but can be exacerbated by pasireotide^61,67^. By contrast, pegvisomant might have a beneficial effect on insulin sensitivity, glucose tolerance and fatty acid metabolism, mainly owing to its consequent suppression of hepatic glucose production^107,108^ (MQ). Close monitoring of glycaemia is recommended for all patients and particularly for those treated with pasireotide (SR). We recommend that hyperglycaemia is treated promptly (SR).

Patients with acromegaly are at increased risk of colorectal adenomatous polyps and colorectal cancer^109^. However, a conclusive association between the frequency of colonoscopic surveillance and cancer-specific mortality in patients with acromegaly but not concurrent high-risk factors, such as known polyps or a family history of polyps, has not been shown^110^ (MQ). We recommend cancer screening be carried out as recommended for the general population (DR).

## Tumour volume and surgical outcomes

SRLs induce tumour shrinkage via direct and indirect antiproliferative effects^111^. Approximately half of patients show considerable tumour reduction within the first few months of treatment with primary or adjuvant SRLs (MQ); these changes typically, but not necessarily, correlate with biochemical control^46,47,112–115^ (LQ). Pasireotide might exert a greater effect on tumour control than octreotide and lanreotide^66^ (LQ). Patients with acromegaly owing to genetic causes, such as *AIP* mutations and X-linked acrogigantism, might exhibit larger tumours that could be less responsive to therapy than tumours in patients with sporadic acromegaly^116–118^ (VLQ).

Although preoperative treatment with SRLs can reduce tumour size and improve surgical cure rates in patients with macroadenomas^119,120^ (LQ), routine use of SRLs for this purpose is not recommended, as evidence for a benefit on postoperative outcomes remains unclear^121^ (DR).

Increased tumour growth associated with pegvisomant therapy has been reported, particularly in patients who switch from an SRL to pegvisomant^122,123^ (LQ). However, large observational studies carefully examining reported cases found it to be rare^75^ and not more frequent than in patients on SRL therapy (MQ); furthermore, the mechanisms underlying the effect remain unclear. Nevertheless, the possibility of tumour growth with pegvisomant should be taken into consideration when selecting treatment, and ongoing imaging surveillance is advised for patients with notable residual tumour who are treated with pegvisomant (SR) (Fig. 1). Data on the effects of cabergoline on tumour volume are insufficient to form a recommendation^58^ (VLQ).

Imaging frequency to assess tumour volume should be individualized to each patient. We recommend that baseline tumour size and location, current medical therapy and its presumed effect on tumour mass, as well as persistent activity or biochemical relapse of the disease, should all be considered (DR).

## Factors in pharmacological choices

Although the initial therapy choice will largely be driven by tumour and biochemical characteristics, we recommend that other patient-specific and disease-specific factors should be considered to appropriately individualize the therapeutic approach^45^ (DR). For example, although reduction of acromegaly disease activity might lead to improvements in insulin sensitivity, worsening of hyperglycaemia can occur during therapy, largely owing to inhibition of insulin secretion by SRLs (MQ). This factor is particularly relevant with the use of pasireotide but might also be relevant for the use of first-generation SRLs (LQ). Thus, for patients with impaired glucose metabolism and/or for those who experience worsening hyperglycaemia on SRL therapy, we recommend that pegvisomant or cabergoline can be considered as alternative options (DR). We also recommend that hyperglycaemia owing to acromegaly-directed therapy should be managed to aggressively control glucose levels (SR).

We recommend that tumour location (that is, proximity to the optic chiasm) as well as tumour size and the presence of local effects of the tumour mass (such as visual field defects and headache) should be used to determine treatment choice on the basis of the likely effect of therapy on tumour volume (SR).

Well-studied clinical and pathological predictors of responsiveness should also be considered. Tumours showing dense GH granulation on pathology demonstrate greater responsiveness to first-generation SRL therapy than sparsely granulated adenomas^45,124,125^ (LQ), whereas T2-hyperintense tumours are less likely to respond to SRL therapy than other tumours^48,126^ (LQ).

Other pathological markers, including immunohistochemistry to assess somatostatin receptor type 2 (SST2) and SST5 expression as well as dopamine receptor status^124,127^, might be useful for individualizing treatment decisions (VLQ). These markers, however, require further prospective validation and harmonization of scoring systems to determine a personalized approach to use, as they are not approved for routine laboratory use and still remain investigational^128^.

## Proposed place in the guidelines

### First-line medical therapy

Surgical resection of the pituitary adenoma by an experienced neurosurgeon is recommended where possible and represents the optimal opportunity for cure (SR). Primary medical therapy with an SRL might be considered if surgery is contraindicated or if a poor likelihood of success is expected owing to patient-specific and/or tumour-specific factors (DR).

For patients with persistent disease after surgery, a first-generation long-acting SRL is recommended as first-line medical therapy (SR). The choice between octreotide LAR and lanreotide autogel is determined by availability, convenience of administration and patient preference (DR). Cabergoline can be attempted as a first-line medical therapy in patients with acromegaly and mildly elevated levels of IGF1 of <2.5 times the upper limit of normal (DR).

### Second-line medical therapy

We recommend that additional therapies are necessary when first-line medical therapy is not successful in normalizing levels of IGF1 (SR) (Fig. 1). For patients who achieve partial response (a decrease in GH and/or IGF1 ≥50%) after using a long-acting first-generation SRL as first-line medical therapy, we recommend that increasing the dose of the SRL and/or increasing the dose frequency of lanreotide autogel should be attempted (DR). We recommend the addition of cabergoline to continued SRL treatment when levels of IGF1 remain modestly elevated during SRL administration. If a tumoural remnant is surgically resectable, which would enable a considerable decrease in tumour mass, a second surgical intervention might be proposed before re-initiating SRL treatment.

If biochemical control is not achieved after administering the maximal dose of first-generation SRL, we recommend that treatment should be individualized on the basis of the presence or absence of clinically relevant residual tumour and impaired glucose tolerance (SR). If a clinically relevant residual tumour that is unsuitable for resection is present, we recommend that patients should be switched from first-generation SRL to pasireotide LAR (DR); if severe hyperglycaemia occurs, patients should be switched to pegvisomant (DR). However, if there is pre-existing clinically relevant impaired glucose metabolism, patients should be switched from first-generation SRL to pegvisomant (DR). If there is clinically relevant residual tumour and pre-existing impaired glucose metabolism, maintaining first-generation SRL and adding pegvisomant is recommended (DR).

### Additional considerations

If biochemical control is not achieved after second-line therapy, stereotactic radiosurgery or surgical intervention or reintervention should be reconsidered, as appropriate (SR). Use of temozolomide should be limited to patients with unusually aggressive or proven malignant pituitary tumours^129^. In such cases, close cooperation with a neuro-oncologist is advisable (DR).

## Conclusions

Our recommendations for management of acromegaly have markedly changed since the previous consensus published in 2014 (ref.^4^). With the availability of pasireotide LAR, patients now have more treatment options and are more likely to achieve biochemical control. At the same time, clinicians should be vigilant about tailoring treatment approaches to account for the full clinical disease spectrum, taking into account biochemical control rates as well as tumour profile and glucose metabolism. Further study of current and emerging agents will help to better define the patient populations most likely to benefit from each treatment strategy and to tailor acromegaly treatments to individual patient needs.
